# Idiopathic mast cell activation syndrome and radiation therapy: a case study, literature review, and discussion of mast cell disorders and radiotherapy

**DOI:** 10.1186/s13014-019-1434-6

**Published:** 2019-12-09

**Authors:** Robin E. Landy, William C. Stross, Jackson M. May, Tasneem A. Kaleem, Timothy D. Malouff, Mark R. Waddle, Laura A. Vallow

**Affiliations:** 1 Florida State University College of Medicine, 1115 W Call St, Tallahassee, FL 32304 USA; 2 Department of Radiation Oncology, Mayo Clinic, 4500 San Pablo Road S, Jacksonville, FL 32224 USA

**Keywords:** Radiation therapy, Mast cell activation syndrome, Allergy, Breast cancer, Toxicity

## Abstract

**Background:**

Mast Cell Activation Syndrome (MCAS) is classified as an idiopathic mast cell disorder where inconsistent or unknown triggers release inflammatory mediators and cause a constellation of symptoms. Studies demonstrate mast cells increase histamine, tryptase, and inflammatory cytokine expression following ionizing radiation. Additionally, there are cases of cutaneous mastocytosis developing within the initial radiation field suggesting mast cells play a role in local tissue reactions. Literature is sparse on radiation induced toxicity in patients with mast cell disorders.

**Case presentation:**

A 62 year old female patient with a history of MCAS received breast conservation therapy for invasive lobular carcinoma of the left breast initially AJCC 7th Stage IIB, pT3 pN0 M0. The patient underwent external beam radiotherapy (EBRT) and received 4500 cGy to the left whole breast, followed by a 1000 cGy boost to the lumpectomy cavity. She developed grade 1 radiation dermatitis. Two years later she progressed distantly and received stereotactic body radiation therapy to a lumbar vertebrae lesion to a dose of 2400 cGy in a single fraction. She developed no in-field dermatologic or systemic flare in her MCAS symptoms during radiation therapy.

**Conclusions:**

To our knowledge there are no reported cases in the literature of patients diagnosed with MCAS or other idiopathic mast cell disorders undergoing radiation therapy. Idiopathic mast cell disorders such as MCAS and primary mast cell disorders alike should not be considered a contraindication to treatment with EBRT. This patient population appears to tolerate treatment without systemic flares in symptoms.

## Background

Mast cells are important components of inflammation, allergy, and immune responses and concentrate in mucosal tissues, such as the oronasopharynx, skin, lungs, and gastrointestinal tract [1]. Allergens cause downstream release of histamine, tryptase, leukotrienes, prostaglandins, and other cytokines which interact with end-organs to cause a variety of physiologic responses and symptoms [2]. Mild symptoms of mast cell mediator release include rhinitis, flushing, skin reactions, headaches, and gastrointestinal complaints [3, 4]. More severe allergic reactions include hypotension, airway compromise due to bronchoconstriction, and anaphylaxis [5, 6].

Mast cell disorders are subdivided into primary, secondary, and idiopathic categories with specific diagnostic criteria as detailed in the Journal of Allergy and Clinical Immunology [3]. Primary mast cell disorders include systemic mastocytosis and cutaneous mastocytosis, in which mast cells are morphologically abnormal or abnormally proliferating. Secondary mast cell disorders are due to underlying allergic, autoimmune, or neoplastic processes where known allergens, complement, or circulating cytokines signal mast cell mediator release. Disorders of mast cell mediator release from inconsistent or unknown triggers are classified under the idiopathic mast cell disorder category. The proposed diagnostic criteria by Akin et al., for Mast Cell Activation Syndrome (MCAS) are as follows: 1) symptomatic flares impacting two or more organ systems, 2) documented elevation of mast cell activators during flares on two separate occasions, 3) response to anti-histamine and anti-leukotriene medications, and 4) absence of a primary or secondary causes for mast cell activation. Epidemiology is not available on the incidence of this specific disorder as this classification of disorder was made recently. Individualized symptomatic control is the standard treatment for MCAS [4]. Mild-to-moderate symptoms are treated with prophylactic or episodic anti-histamine, anti-leukotriene, and cromolyn medications. Patients with severe reactions should be prescribed epinephrine auto-injectors [3, 4, 7].

Patients with bone lesions from primary mast cell disorders have been treated with external beam radiation therapy [EBRT] without systemic flares in symptoms, such as skin reactions outside of the radiation field, gastrointestinal complaints, bronchospasm, anaphylaxis or hypotension [8–10]. To our knowledge, there have been no reported cases of patients with idiopathic mast cell disorders undergoing radiation therapy. The rate and clinical impact of local skin toxicity and potential systemic reaction to radiation therapy is unknown.

## Case presentation

The patient initially presented to our institution at age 47 for evaluation in the rheumatology department. For years the patient had periodical episodes of flushing, blurred vision, pruritus, hives, periorbital edema, globus sensation, dysphonia, sinus drainage, cough, abdominal cramping, and diarrhea. She associated this constellation of symptoms to exposure to environmental triggers including increased heat, perfumes, excessive exposure to sunlight, certain foods, and stress. Her comorbid past medical history included hypertension, hyperlipidemia, gastroesophageal reflux disease, Ehlers-Danlos syndrome, postural orthostatic tachycardia syndrome, depression, and multiple sclerosis. Medications at presentation included aspirin, diltiazem, clonidine, simvastatin, omeprazole, sucralfate, bupropion, and gabapentin. Family history included a brother with pancreatic cancer and multiple first degree relatives with heart disease and osteoarthritis.

Laboratory evaluation demonstrated a trend of persistently elevated urine beta prostaglandin F2-alpha, a known mast cell mediator. Serum N-methylhistamine and serum tryptase levels were normal. The patient eventually underwent bone marrow biopsy, which was negative, ruling out primary mast cell disorders. She was originally diagnosed with “leaky mast cell syndrome” and instructed to use antihistamine medications for symptomatic control and to avoid environmental triggers.

At age 58 her diagnosis was reassessed due to persistent symptoms despite medical therapy and attempted trigger avoidance. Leukotriene E4 and urine beta prostaglandin F2-alpha were confirmed to be elevated during symptom flares which involved multiple organ systems. Primary and secondary mast cell disorders were ruled out with serum protein electrophoresis showing no monoclonal protein spike and gene mutation analysis showed no mutation in c-KIT Asp816Val. Additional serum and urine metabolites were tested and found to be normal, ruling out carcinoid and serotonin syndromes. Auto-antibody panels, erythrocyte sedimentation rate, and C-Reactive Protein levels were not consistent with an autoimmune disorder. The patient was ultimately diagnosed with MCAS by the previously mentioned diagnostic criteria. She continued daily antihistamine therapy, trigger avoidance, and dietary modifications for symptomatic control.

At age 62 the patient returned to our institution for an abnormal screening mammogram demonstrating a suspicious mass in the 2 o’clock position in the left breast. Further diagnostic imaging confirmed a 5.6 cm mass with no pathologic adenopathy or distant metastatic disease. Ultimately the patient underwent left partial mastectomy and axillary sentinel lymph node biopsy. Final pathology revealed 5.5 cm tumor containing grade 2 invasive lobular carcinoma that was estrogen receptor (ER) and progesterone receptor (PR) positive, and Her2/neu negative. Two axillary lymph nodes were negative for malignancy confirming Stage IIB, pT3 pN0 M0, according to AJCC 7th Edition.

The patient declined the recommended adjuvant chemotherapy due to concern of side effects from her comorbid conditions. The patient did proceed to post-operative external beam radiation therapy and received 4500 cGy to the left whole breast in 25 fractions, followed by a 1000 cGy boost to the lumpectomy cavity over 5 fractions. The patient developed grade 1 radiation dermatitis within the treatment field. She was without a systemic flare in her MCAS. Seven weeks after radiation was completed, clinical exam revealed mild hyperpigmentation within the treatment field. The patient denied any local skin reactions such as pruritus, hives, or rash within the radiation field, alone or in association with symptom flares. The patient proceeded to Letrozole hormonal therapy and tolerated this medication well. Under the care of rheumatology her MCAS was subsequently managed with maintenance Montelukast for pulmonary symptoms, Ketotifen eye drops for ocular symptoms, as needed Loperamide for diarrhea, Dicyclomine for abdominal cramping, and Fexofenadine maintenance antihistamine therapy.

At age 64 the patient developed low back pain and radicular symptoms and was ultimately biopsy confirmed to have metastatic progression to the first lumbar vertebrae. She received stereotactic body radiation therapy to a dose of 24 Gy in 1 fraction. She developed temporary increased frequency of stools in the week following SBRT but had no other reported toxicity or any multi-organ symptom manifestations that were typical for her MCAS flares. Her radicular symptoms improved following radiation, but she still required opioid analgesia for back pain on an as needed basis. She transitioned to systemic therapy with exemestane and continues to follow up with her multi-disciplinary team in oncology and rheumatology.

## Discussion and conclusions

Ex-vivo and in-vivo studies demonstrate human mast cells increase histamine, tryptase, and inflammatory cytokine expression following ionizing radiation [11, 12]. These mast cell mediators serve an integral role in downstream fibroblast inflammatory response, leading to cutaneous radiation reactions [11, 13]. There are reported cases of patients developing cutaneous mastocytosis and urticarial following radiation therapy, with the initial dermatologic lesions arising within the radiation field [14–17]. These studies and clinical cases suggest mast cells play a key role in local tissue reactions, however less is known regarding the systemic effects of mast cell mediator release stemming from local radiation treatment.

To our knowledge there are no reported cases in the literature of patients diagnosed with MCAS or other idiopathic mast cell disorders undergoing radiation therapy. There are reported cases of patients with primary mast cell disorders having undergone palliative radiation therapy for painful bone lesions. Table 1 summarizes the lesion locations, fractionation schedules, and clinical outcomes of these cases [8–10, 18]. Most of the patients had good clinical response with regards to pain and functional status, and all were without clinical flare in symptoms related to their underlying systemic mastocytosis. In select cases, plasma histamine levels were trended and showed no significant change throughout the radiation course.

**Table 1:** Summary of patients with primary mast cell disorders who were treated with external beam radiation therapy. T = Thoracic, L = Lumbar, SI = Sacroiliac, EBRT = external beam radiation therapy

Author, Date	Location	Dose and Fraction	Outcome
Janjan, 1992	Extensive disease in the T-L spine and bilateral SI joints (T8-SI joints treated)	30 Gy in 11 fractions, split course	Improvement in pain. No systemic reaction. Trended serum histamine levels during EBRT demonstrated no elevation.
Johnstone, 1994	T12/L1 cord compression and painful left shoulder lesion	30 Gy in 10 fractions, both sites	Moderate improvement in pain. No systemic reaction.
Johnstone, 1994	Bilateral lower extremity lesions	30 Gy in 15 fractions	Marked pain relief. Mild thrombocytopenia. No systemic reaction.
Harrison, 1994	Multifocal disease: T7–11, L2–5, and SI joints.	20 Gy in 5 fractions	Transient improvement in pain and increased mobility prior to disease progression. No systemic reaction.
Hesselmann, 2001	Multifocal disease: hands, knees, left humerus, right shoulder.	40 Gy in 16–20 fractions	No clinical or hematologic adverse events. No systemic reaction.

Histamine serves an important role in local tissue response to radiation therapy as demonstrated in ex-vivo and in-vivo studies. The results from our patient’s experience and those treated for bone pain secondary to systemic mastocytosis demonstrate no clinically apparent increase in systemic symptoms or laboratory testing evidence of mast cell mediator release on serologic testing during EBRT. Authors recommend multi-disciplinary approach in treating patients with idiopathic mass cell disorders and malignancy. Though the data is limited, patients with idiopathic mass cell disorders and primary mast cell disorders alike appear to tolerate external beam radiation therapy well, without systemic flare in symptoms. These disorders should not be considered a contraindication to treatment with radiation therapy. In patients with severe flare symptoms of anaphylaxis, angioedema, or hypotension clinicians can consider trending mast cell mediator levels throughout radiation treatments.

## Data Availability

Not applicable. All data generated or analyzed during this study are included in this published article and its supplementary information.

## Abbreviations

EBRT: External beam radiotherapy
ER: Estrogen receptor
L: Lumbar
MCAS: Mast cell activation syndrome
PR: Progesterone Receptor
SI: Sacroiliac
T: Thoracic
